# The Transcriptome of *Verticillium dahliae* Responds Differentially Depending on the Disease Susceptibility Level of the Olive (*Olea europaea* L.) Cultivar

**DOI:** 10.3390/genes10040251

**Published:** 2019-03-27

**Authors:** Jaime Jiménez-Ruiz, María de la O Leyva-Pérez, Carmen Gómez-Lama Cabanás, Juan B. Barroso, Francisco Luque, Jesús Mercado-Blanco

**Affiliations:** 1 Center for Advanced Studies in Olive Grove and Olive Oils, Department of Experimental Biology, University of Jaén, 23071 Jaén, Spain; ruiz@ujaen.es (J.J.-R.); Maria.Leyva@teagasc.ie (M.d.l.O.L.-P.); jbarroso@ujaen.es (J.B.B.); fjluque@ujaen.es (F.L.); 2 Department of Crop Protection, Institute for Sustainable Agriculture, Agencia Estatal Consejo Superior de Investigaciones Científicas (CSIC), Campus ‘Alameda del Obispo’, Avenida Menéndez Pidal s/n, 14004 Córdoba, Spain; cgomezlama@ias.csic.es

**Keywords:** defoliating pathotype, effector, pathogenicity, RNA-seq, susceptibility, vascular pathogen, *Verticillium dahliae* transcriptome, Verticillium wilt of olive

## Abstract

Among biotic constraints affecting olive trees cultivation worldwide, the soil-borne fungus *Verticillium dahliae* is considered one of the most serious threats. Olive cultivars display differential susceptibility to the disease, but our knowledge on the pathogen’s responses when infecting varieties differing in susceptibility is scarce. A comparative transcriptomic analysis (RNA-seq) was conducted in olive cultivars Picual (susceptible) and Frantoio (tolerant). RNA samples originated from roots during the first two weeks after inoculation with *V. dahliae* defoliating (D) pathotype. *Verticillium dahliae* mRNA amount was overwhelmingly higher in roots of the susceptible cultivar, indicating that proliferation of pathogen biomass is favored in ‘Picual’. A significant larger number of *V. dahliae* unigenes (11 fold) were only induced in this cultivar. Seven clusters of differentially expressed genes (DEG) were identified according to time-course expression patterns. Unigenes potentially coding for niche-adaptation, pathogenicity, virulence and microsclerotia development were induced in ‘Picual’, while in ‘Frantoio’ expression remained negligible or null. *Verticillium dahliae* D pathotype transcriptome responses are qualitatively and quantitatively different, and depend on cultivar susceptibility level. The much larger *V. dahliae* biomass found in ‘Picual’ roots is a consequence of both host and pathogen DEG explaining, to a large extent, the higher aggressiveness exerted over this cultivar.

## 1. Introduction

The soil-borne, hemibiotrophic pathogen *Verticillium dahliae* Kleb. infects hundreds of plant species, causing vascular diseases (Verticillium wilts) in many high-value crops worldwide [1,2,3]. A broad host range, the production of dormant, long-term enduring structures (i.e., microsclerotia) in soil and infected plant debris, and the systemic nature of infections are factors explaining the extreme difficulty for the effective control of this pathogen [4]. *Verticillium dahliae*, and especially the highly virulent defoliating (D) pathotype (lineage 1A) [5], severely affects cultivated olive (*Olea europaea* L. subsp. *europaea* var. *europaea*) in many regions where it is cultivated, particularly in the Mediterranean Basin where this tree species has unquestionable economic and social importance [6,7,8]. Since no control measure has proved to be successful when implemented individually, an integrated disease management strategy to control Verticillium wilt of olive (VWO) is recommended [9].

The olive–*V. dahliae* interaction has been investigated by different methodological approaches (e.g., microscopy, histochemistry, transcriptomics, etc.), enabling a good knowledge on relevant aspects of this pathosystem such as the pathogen’s colonization process [10], the effect of specific abiotic factors (i.e., soil temperature) on the host response to VWO [11], the triggering of defence-related systemic responses upon *V. dahliae* infection [12], and histological and biochemical responses taking place in host tissues during pathogen’s attack [13,14,15,16,17].

Our understanding of plant–microbe interactions is rapidly increasing by whole-genome and whole-transcriptome sequencing approaches. In the particular case of *V. dahliae*, RNA-seq has been used to analyze gene expression in microsclerotia e.g., [18,19], or to study the interaction of the pathogen with hosts such as cotton [20,21], tobacco [22], tomato [23] and olive [24].

Whole-transcriptome studies aiming to unravel the underlying genetic bases of plant–microbe interactions are normally focused on the transcriptome of one of the partners of the interaction, either the host or the pathogen as in the examples mentioned above. Only in a few cases, global transcriptomic responses are studied for both organisms during their interaction e.g., [24]. In this latter study, a co-transcriptome for olive and *V. dahliae* D pathotype was generated. The olive transcriptome (‘Oleup’) was further refined and augmented (generating the so-called ‘PicFra’ transcriptome) after a comparative transcriptomic study aiming to disentangle early olive responses related to tolerance/susceptibility upon *V. dahliae* infection [25]. Remarkably, comparative transcriptomic studies dealing with interactions involving a pathogen and host genotypes differing in susceptibility level are overwhelmingly, if not exclusively, focused on gene expression changes occurring in the host. Indeed, an increasing body of knowledge is available on global genetic responses of host genotypes showing differential susceptibility to the attack of pathogens such as *Phytophthora nicotianae* in *Citrus* spp. [26], ‘*Candidatus Liberibacter asiaticus*’ in *Citrus hystrix* [27], *Fusarium fujikuroi* in rice [28], *Xanthomonas perforans* Race T3 in tomato [29], or *Xylella fastidiosa* in olive [30], just to name a few recent examples. For *Verticillium* spp., comparative transcriptomics have provided relevant information on the genetic basis underlying host tolerance/susceptibility in the pathosystems *V. dahliae*-olive [25] or *V. nonalfalfae*-hop (*Humulus lupulus* L.) [31]. However, whole transcriptome responses of a pathogen during the interaction with host genotypes which display differential susceptibility are rather unknown.

In a previous study, the *V. dahliae* transcriptome ‘Vedah’ was generated in addition to the above-mentioned olive transcriptome [24]. It consisted of nearly 38,000 V*. dahliae* unigenes (more than 52,000 isoforms/transcripts). Furthermore, differentially expressed genes (DEGs) of *V. dahliae* overexpressed in infected olive roots of the susceptible cultivar (cv.) Picual were mostly related to general metabolism. However, the induction of genes potentially coding for the reactive oxygen species (ROS) defence response was also observed. Interestingly, this response took place earlier in *V. dahliae* than in olive roots, where a ROS response induction was also detected [24]. It thus seemed that a ROS protective response was quickly induced in *V. dahliae* hyphae upon interaction with olive roots, whilst root tissues developed a response to the ROS stress at later stages. Moreover, the response to ROS stress was much stronger in the susceptible cv. Picual than in tolerant ‘Frantoio’ plants, as recently demonstrated by Leyva-Pérez and co-workers [25]. Thus, a still open question is whether the ROS protective response in *V. dahliae* also differs depending on the VWO tolerance level of the cultivar. Furthermore, *V. dahliae* DEGs coding for four chitin-binding proteins likely involved in inactivation of the plant endochitinase PR4 protein, two Ace1-like DEGs that could indirectly mediate avirulence [32], and the MiniChromosome Maintenance (*mcm*1) gene, described as a virulence factor in *V. dahliae* [33], were also found to be expressed during the interaction [24]. It would be interesting to assess whether these and others pathogen effectors, as well as pathogenicity and virulence factors and other genes relevant for colonization and infection show differential expression patterns depending on the infected olive genotype.

While analysis of whole-transcriptome olive responses in roots showed clear differences depending on the VWO susceptibility/tolerance level of the cultivar, our knowledge on whether the transcriptome of *V. dahliae* also responds differentially depending on this host phenotype is null. Within this framework, and considering the above-mentioned antecedents, we aimed here to unravel *V. dahliae* transcriptome responses upon infection of susceptible (‘Picual’) and tolerant (‘Frantoio’) olive cultivars. We will examine global transcriptomic responses from the pathogen’s perspective and from the same experimental scenario from which host responses were previously scrutinized. The objectives of this work are: (i) to compare *V. dahliae* (D pathotype, linage 1A) whole-transcriptome responses and to assess whether time-course differences occur upon interaction with olive cultivars displaying different tolerance to the pathogen, and (ii) to identify differential expression of ad hoc targeted *V. dahliae* genes related to effectors, pathogenicity, virulence and niche adaptation, in order to determine whether the pathogen uses different strategies to infect olive cultivars differing in susceptibility to VWO. The hypothesis to-be-tested is that differences in tolerance are not only due to the host responses but also to an overall differential response of the *V. dahliae* transcriptome.

## 2. Materials and Methods

### 2.1. *Verticillium dahliae*–olive bioassays

The *V. dahliae* isolate V937I [34], representative of the highly-virulent, D pathotype (linage 1A) [35] was used. The V937I inoculum (conidia suspension) was prepared as described by Jiménez-Ruiz and co-workers [24]. All tissue samples originated from bioassays performed and described in our previous works [24,25]. These experiments were carried out to assess, at the whole-transcriptome level, the interaction under non-gnotobiotic conditions between olive cultivars differing in susceptibility to VWO and the D pathotype of *V. dahliae*. Therefore, for this present study, the same infected tissue samples generated by Leyva-Pérez and co-workers [25] were analysed. Briefly, olive plants (cvs. Frantoio and Picual; four-month-old) obtained from a commercial nursery located at Córdoba province (southern Spain) were inoculated by immersing their roots systems in a conidial suspension (1 × 10^7^ conidia mL^−1^ for 30 min) of the isolate V937I as previously described [36]. ‘Frantoio’ is tolerant to VWO, while ‘Picual’ is very susceptible to D isolates of *V. dahliae* [9,12,25]. Inoculated plants were then individually transplanted into polypropylene pots filled-in with an ad hoc prepared autoclaved sandy substrate [37]. Culturing conditions were: random distribution of pots in a growth chamber adjusted to 24 °C (day)/21 °C (night), 60% relative humidity, 14-h photoperiod of fluorescent light (360 µE m^−2^ s^−1^) during 15 days (for RNA sampling) or two months (for VWO symptoms observation). The photoperiod was gradually increased to alleviate plant stress after manipulation, inoculation and transplanting [12,24]. Roots of each plant were harvested at 0, 2, 7 and 15 days (three plants/time point) after V937I inoculation (DAI). Tissue samples were immediately frozen in liquid nitrogen and kept at −80 °C until total RNA extraction. A group of ‘Picual’ and ‘Frantoio’ *V. dahliae*-inoculated and non-inoculated plants were kept to verify actual VWO symptoms development.

### 2.2. Preparation of RNA Samples and Sequencing

Groups of randomly-selected plants (3 for each cultivar) per treatment (inoculated and non-inoculated) and time point (see above) were defined in order to reduce plant-to-plant variability, as described by Leyva-Pérez and co-workers [25]. Total RNA samples from roots sampled at the time-points indicated above were extracted using a ‘Spectrum Plant Total RNA kit’ (Sigma-Aldrich, St Louis, MO, USA), following the manufacturer’s indications. Contaminant DNA was removed with on-column DNase digestion (Roche, Basel, Switzerland). The Agilent 2100 bioanalyser (Agilent Technologies, Santa Clara, CA, USA) using an RNA 6000 Pico assay kit (Agilent Technologies) was employed for RNA quality tests. Then, equimolar amounts of RNA from each group were pooled, cDNA libraries were prepared, and NGS sequencing was performed at Sistemas Genómicos S.L. (Paterna, Valencia, Spain) using an Illumina HiSeq 1000 sequencer. Two replicates per sample were sequenced on different lanes in the flow cell [25].

### 2.3. RNA-seq Data Pre-Processing

Data pre-processing was carried-out as described by Leyva-Pérez and co-workers [25]. Briefly, Fastqmcf [38] was used to first pre-process raw Illumina RNA-seq reads, discarding primers and reads with adaptors, unknown nucleotides and poor-quality or short-length reads, thereby increasing the Qscore to >30 for all the libraries and length >50 bp (Q30L50). A thorough quality control of sequencing was carried out twice with FastQC software (Version 0.10.1) (University of New South Wales, UNSW, Sydney, Australia) [39] to provide a summary and compare statistics files, before and after pre-processing.

### 2.4. Gene expression and Differentially Expressed Genes

Gene expression was carried out with RNA-Seq by Arraystar Lasergene 13, using a 95% False Discovery Rate (FDR) and validated by Jiménez-Ruiz and co-workers [24]. Reference transcriptome was obtained from the *V. dahliae* (‘Vedah’) transcriptome consisting of 37,425 unigenes (52,119 isoforms/transcripts) described in Jiménez-Ruiz and co-workers [24]. The most specific Gene Ontology (GO)-term-enriched analysis was performed with Blast2GO [40]. Results obtained by the RNA-seq analysis were already validated by qPCR, and details have been previously reported [24,25].

### 2.5. Data and Materials Availability

The Illumina sequenced read data reported in this study have been deposited in the National Center for Biotechnology Information (NCBI) Sequence Read Archive and are available under the following Accession Numbers: SRR5330848, SRR5330847, SRR5330842, SRR5330843, SRR5330844, SRR5330845 [25], and SRR6760519, SRR6760520 (this work). The Transcriptome project has been deposited under the accession number PRJNA378602.

## 3. Results

### 3.1. Global mRNA Amount of *Verticillium Dahliae* D pathotype is Strikingly Higher in ‘Picual’ (VWO-Susceptible) Than in ‘Frantoio’ (VWO-Tolerant) Roots

**Figure 1:**
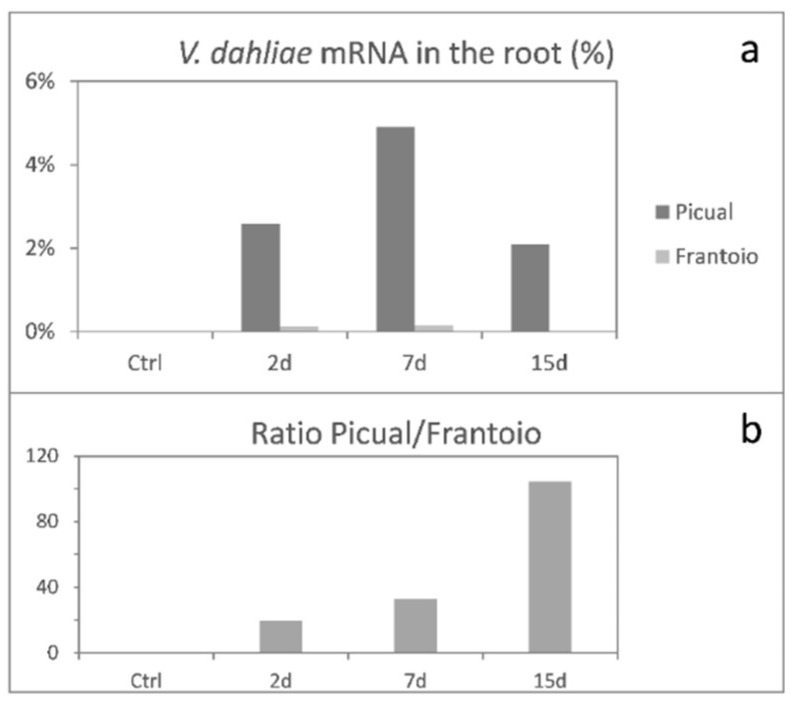
Accumulation of *Verticillium dahliae* transcripts in the roots of inoculated plants during early stage of infection in the susceptible variety ‘Picual’ and tolerant ‘Frantoio’. The percentage of the total paired-end reads that aligned with *V. dahliae* genes is represented for each time point. Only the paired-end reads that aligned both ends unequivocally at the correct distance with fungal genes are represented (**a**). Ratio of Picual/Frantoio percentage of reads is represented for each time point (**b**).

In order to measure *V. dahliae* growth during the olive root infection process, the percentage of total paired-end reads unequivocally aligning both ends with *V. dahliae* genes and at the correct distance was obtained for each time point (Figure 1a). As mRNAs are short half-life molecules, the RNAseq data of fungal mRNA paired-end reads present on/in root tissues at a given sampling time-point constitute a direct estimation of the amount of metabolically active fungal biomass. Maximum accumulation of *V. dahliae* mRNA was detected at 7 DAI in both cultivars. Remarkably, the percentage of pathogen transcripts was a lot higher in ‘Picual’ than in ‘Frantoio’ roots, indicating that the VWO-susceptible cultivar significantly favored the proliferation of *V. dahliae* biomass in contrast to the mycelial growth observed in ‘Frantoio’ roots. Moreover, the Picual/Frantoio ratio of the percentage of reads increased over time (Figure 1b).

### 3.2. An Overwhelming Number of *Verticillium Dahliae* Unigenes Are Only Induced in Roots of the Susceptible Cultivar Picual

Whole-transcriptome responses of *V. dahliae* D pathotype clearly differed between ‘Picual’ and ‘Frantoio’ roots. Furthermore, RNA-seq analysis of *V. dahliae* transcripts showed a much higher number of unigenes induced in roots of the VWO-susceptible cultivar than in ‘Frantoio’. Thus, using and eightfold change over the almost null data of non-inoculated control plants and a 95% FDR, 30,240 *V. dahliae* unigenes were found to be over expressed in ‘Picual’ roots, but only 5641 in ‘Frantoio’ roots.

**Figure 2:**
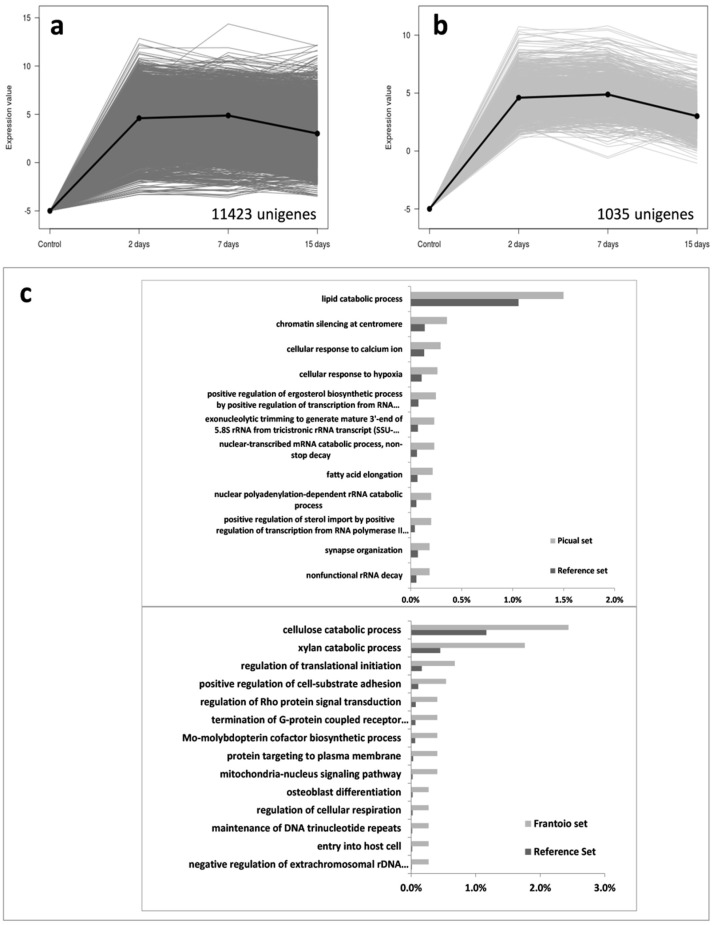
Group 1 unigenes that present a high mRNA level at 2-, 7- and 15-days post-inoculation. Unigenes were selected using an eightfold change and a 95% of False Discovery Rate; mRNA level in ‘Picual’ (**a**) and ‘Frantoio’ roots (**b**); Most specific enriched Gene Ontology (GO) terms of over-expressed genes in *Verticillium dahliae* infecting ‘Picual’ or ‘Frantoio’ roots (**c**).

*Verticillium dahliae* transcript expression profiles obtained during the time-course infection process here under study (0–15 DAI), enabled us to cluster over-expressed unigenes in 7 distinctive groups. Group 1 consisted in pathogen’s unigenes highly induced along the entire period, 11,423 being over-expressed in ‘Picual’ but only 1035 in ‘Frantoio’ (Figure 2). This remarkable difference was not merely quantitative but also concerned the type of *V. dahliae* genes induced in each cultivar. Indeed, while in ‘Picual’ roots the most specific enriched GO terms were diverse and related to different processes such as lipid metabolism, response to calcium or hypoxia, chromatin silencing or other processes related to gene expression, in ‘Frantoio’ roots *V. dahliae* was essentially enriched in unigenes of cellulose and xylan catabolic processes (Figure 2c).

**Figure 3:**
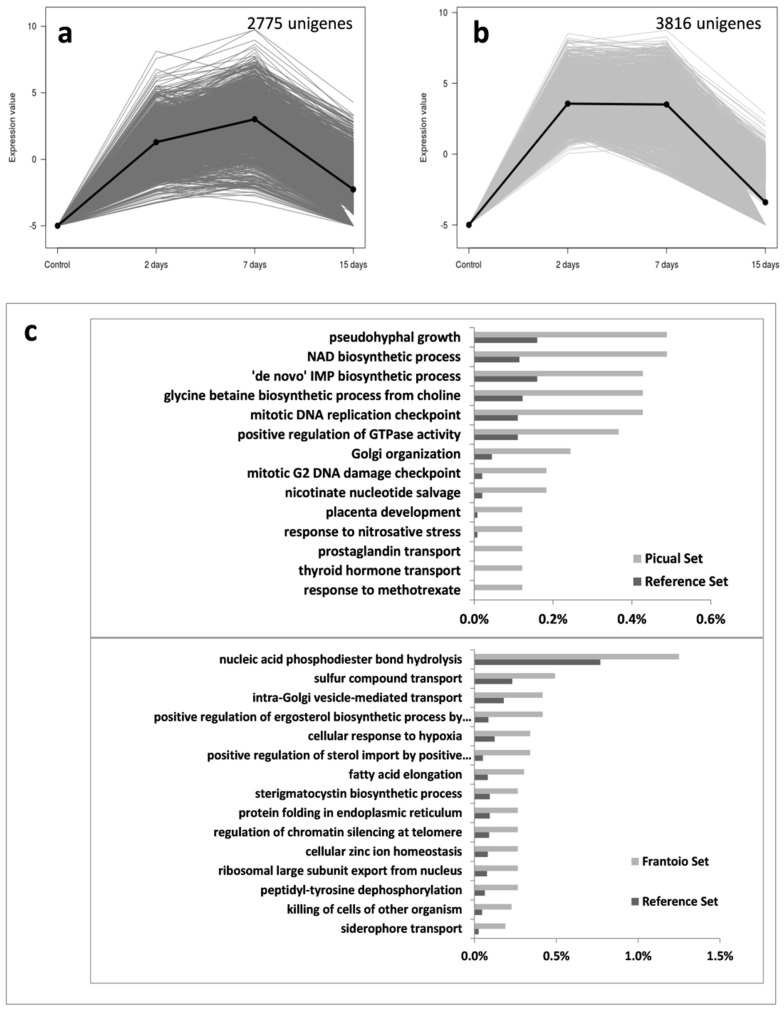
Group 2 unigenes that present a high mRNA level at 2- and 7-days but low at 15-days post-inoculation. Unigenes were selected using an eightfold change and a 95% of False Discovery Rate; mRNA level in ‘Picual’ (**a**) and ‘Frantoio’ roots (**b**); Most specific enriched GO terms of over-expressed genes in *Verticillium dahliae* infecting ‘Picual’ or ‘Frantoio’ roots (**c**).

Group 2 corresponded to *V. dahliae* unigenes that were induced at early moments of the infection (2 DAI), but eventually were repressed at 15 DAI (Figure 3), coinciding with the timing in which fungal biomass decreased in both cultivars (Figure 1). This group consisted of 2775 unigenes in ‘Picual’ and 3816 unigenes in ‘Frantoio’. The most specific enriched GO terms again differed in both olive cultivars. Remarkably, unigenes related to pseudohyphal growth, diverse biosynthetic processes, and mitotic GO terms decayed at 15 DAI in ‘Picual’ roots. This was again consistent with the decrease of fungal growth (i.e., percentage of *V. dahliae* transcripts in roots) observed at that time point (Figure 1). However, most specific enriched GO terms in ‘Frantoio’ roots were related to a wide range of processes, some of them previously found in ‘Picual’ Group 1 (Figure 2c), like cellular response to hypoxia or fatty acid elongation (Figure 3c).

Groups 3, 4 and 5 corresponded to transient (at 2, or 7, or 2/15 DAI, respectively) induction of *V. dahliae* unigenes with no significant feature to be highlighted after conducting most specific enriched GO terms analysis (Figures S1–S3). Fluctuating expression patterns of these transcripts along time are difficult to interpret. The only relevant observation is that, once again, a much higher number of pathogen unigenes were expressed in ‘Picual’ roots compared to that in ‘Frantoio’ roots. Moreover, transient induction at 7 DAI of a large number of genes (5524) was only detected in ‘Picual’ roots (Figure S3).

**Figure 4:**
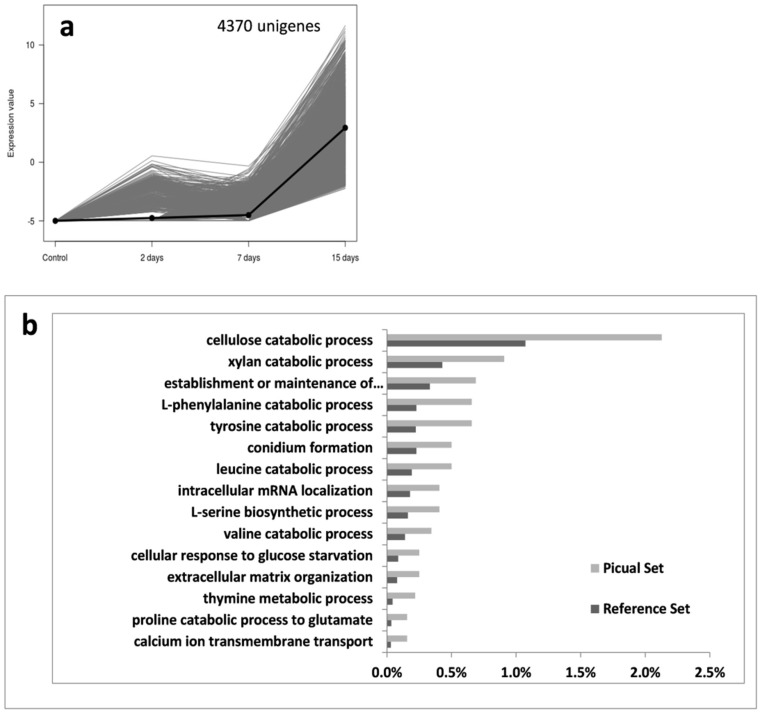
Group 6 unigenes that present a high mRNA level at 15 days but low at 2- and 7-days post-inoculation. Unigenes were selected using an eightfold change and a 95% of False Discovery Rate; mRNA level in ‘Picual’ roots (**a**) and most specific enriched GO terms of over-expressed genes in *Verticillium dahliae* infecting ‘Picual’ roots (**b**).

**Figure 5:**
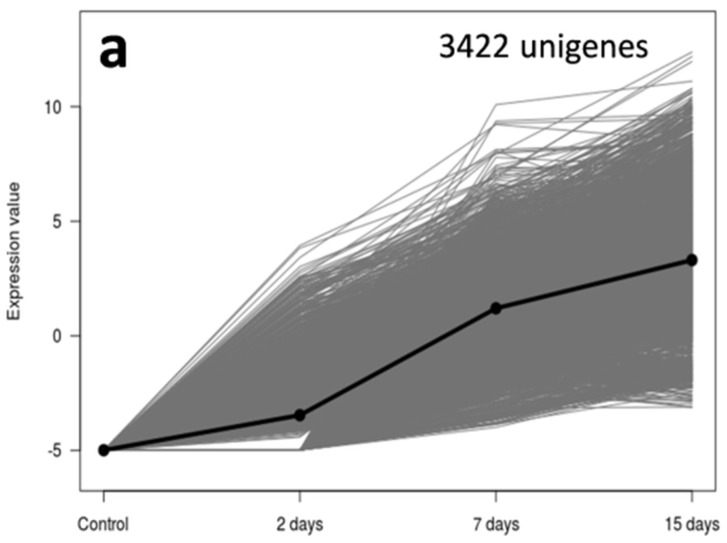

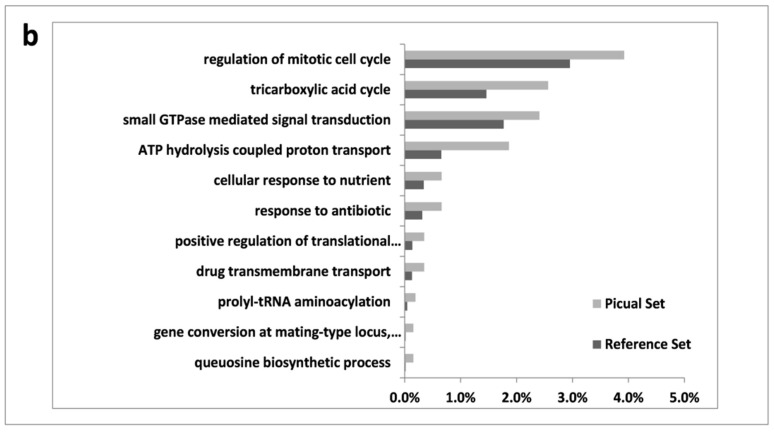
Group 7 unigenes that present a continuous increase of the mRNA level from 2 to 15 days post-inoculation. Unigenes were selected using an eightfold change and a 95% of False Discovery Rate; mRNA level in ‘Picual’ roots (**a**) and most specific enriched GO terms of over-expressed genes in *Verticillium dahliae* infecting ‘Picual’ roots (**b**).

These were also the cases for *V. dahliae* transcripts allocated to Groups 6 and 7. Croup 6 corresponded to unigenes that were induced only in ‘Picual’ at a late stage of the root infection process (15 DAI; Figure 4). This group is comprised by 4370 unigenes, cellulose and xylan catabolic processes being highly represented according to the most specific GO term enrichment analysis (Figure 4b). Interestingly enough, these unigenes were early induced in ‘Frantoio’ roots (i.e., Group 1; Figure 2), but only over-expressed in ‘Picual’ at 15 DAI. In addition, other catabolic processes are induced at this time point, as well as conidia formation (Figure 4b). Finally, Group 7 (only present in ‘Picual’ roots) consisted of 3422 *V. dahliae* unigenes showing a steady increase of gene expression along time (Figure 5). The most specific enriched GO terms of this Group showed prevalence of GO terms related to energy generation, regulation of mitotic cell cycle and cellular response to nutrients.

### 3.3. Targeting *Verticillium Dahliae* Niche Adaptation-Related Genes and Their Differential Expression Patterns in Cultivars Differing in VWO Susceptibility

We examined the time-course gene expression patterns of a number of DEGs related to niche adaptation (some of them also related to other processes; see next epigraphs). To avoid potential misinterpretation of results, particularly considering differences in relative transcripts abundance observed between ‘Picual’ and Frantoio’ (Figure 1), a basal level of log_2_ RPKM (Reads Per Kilobase Million) values >4 was ad hoc established. Therefore, unigenes with expression below this threshold in both varieties were considered as too low expressed to draw any conclusion. This also applies for unigenes described in the following sections.

**Figure 6:**
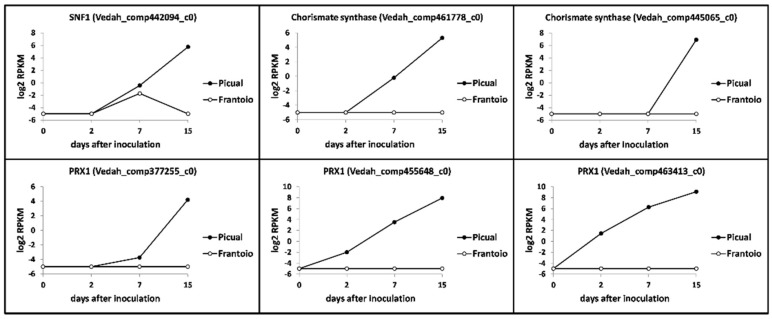
*Verticillium dahliae* unigenes related to niche adaptation and differentially expressed in ‘Picual’ and ‘Frantoio’ roots during infection. Samples were analyzed by RNAseq (see the Results section for details). RPKM, Reads Per Kilobase Million.

Concerning carbon and nitrogen metabolism the most relevant finding concerned one unigene potentially coding for SNF1 (kinase complex involved in catabolite de-repression of carbohydrate active enzymes, CAZyme), that showed a significantly higher expression in ‘Picual’ at 15 DAI compared to the null expression observed in ‘Frantoio’ (Vedah_comp442094_c0) (Figure 6). In addition, two transcripts coding for chorismate synthases (Vedah_comp445065_c0 and Vedah_comp461778_c0) were found to be highly induced in the susceptible cultivar at 15 DAI (Figure 6). Besides, unigenes potentially coding for endoglucanase EG-1 (another CAZyme), SSP1 (Peptidyl-prolyl cis-trans isomerase/Serine/threonine-protein kinase), and CPC1 (cross-pathway control protein 1) detected in the V937-I transcriptome showed either no changes in gene expression (occasionally erratic along time) or an overall higher induction in the susceptible cv. Picual (data not shown), that was assumed to be due to the difference in relative transcripts abundance observed between cultivars (see above; Figure 1).

*Verticillium dahliae* must also cope with other stressing conditions found within the xylem vessels. Unigenes potentially coding for the high-affinity glucose transporter RGT2 were found but no relevant difference in their expression patterns was observed (data not shown). In addition, BLAST analysis did not reveal the presence of a *GT2* (proteobacteria type 2 glucosyltransferase) homolog in V937-I. Stress due to reactive oxidative species (ROS) must also be confronted by *V. dahliae* when proliferating within the xylem. Again, differences were found neither for unigenes potentially coding for catalase-peroxidases nor for proteins of the Thi4 family involved in the biosynthesis of thiazole (precursor of thiamine, vitamin B1), including putative thiamine thiazole synthase transcripts (data not shown). The same could be concluded for unigenes coding for putative α-N-arabinofuranosidase (data not shown). The only remarkable finding related to stress tolerance was the differential up-regulation in ‘Picual’ of four unigenes coding for PRX1 (mitochondrial peroxiredoxin). One of them only showed a transient (2 DAI) significant up regulation in Picual roots (Vedah_comp377200_c0) (data not shown), while the other three (Vedah_comp377255_c0, Vedah_comp455648_c0 and Vedah_comp463413_c0) displayed a steady induction over time reaching maximum expression levels at 15 DAI. In contrast, expression of these unigenes in ‘Frantoio’ remained unaltered and always at a negligible level (Figure 6).

### 3.4. *Verticillium Dahliae* Effector-Coding Unigenes Do Not Show Differential Expression Patterns in ‘Picual’ and ‘Frantoio’ Roots

The disease progress is facilitated by the production of effectors (pathogen-secreted molecules) which either suppress host defenses or protect the pathogen against their action. The expression of unigenes potentially coding for effectors was also investigated. Results showed that, overall, no noticeable differences were found for unigenes coding for LysM, NLP (necrosis- and ethylene-inducing-like proteins), isochorismatase, and secreted, small cysteine-rich proteins found in the V937-I transcriptome, regardless the olive cultivar infected by the D pathotype (data not shown).

### 3.5. *Verticillium Dahliae* D pathotype Unigenes Involved in Pathogenicity, Virulence and Microsclerotia Development Showing Differential Expression Patterns

**Figure 7:**
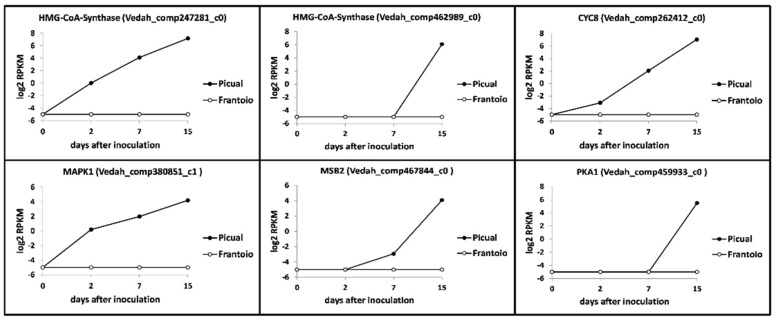
*Verticillium dahliae* unigenes related to pathogenicity, virulence and microsclerotia development and differentially expressed in ‘Picual’ and ‘Frantoio’ roots during infection. Samples were analyzed by RNAseq (see the Results section for details). RPKM, Reads Per Kilobase Million.

*Verticillium dahliae* pathogenicity, virulence and microsclerotia production pathways are often interconnected. Relevant results on unigenes potentially coding for these traits and that showed significantly-different expression patterns are now summarized. Two transcripts coding for hydroxymethyl-glutaryl (HMG)-CoA synthase (Vedah_comp247281_c0 and Vedah_comp462989_c0) were significantly up-regulated at late times after inoculation in the susceptible cultivar, while remained at null expression level in ‘Frantoio’ (Figure 7). Several unigenes potentially coding for CYC8 (glucose repression mediator protein) were found in the V937-I transcriptome. Only one of them (Vedah_comp262412_c0) showed a steady increased expression over time in ‘Picual’, reaching a log_2_ RPKM value > 6 at 15 DAI. In contrast, no expression was detected for this unigene in ‘Frantoio’ roots (Figure 7). The same behaviour was observed for a transcript coding for a mitogen-activated protein kinase MAPK1 (Vedah_comp380851_c1) (Figure 7). A transcript (Vedah_comp467844_c0) coding for a MSB2 protein (transmembrane mucin highly conserved in the MAPK signal pathway) was also found showing a clearly different expression pattern in ‘Picual’ (log_2_ RPKM value > 4 at 15 DAI) compared to that observed in ‘Frantoio (null expression) roots (Figure 7). Finally, several unigenes potentially coding for cAMP-dependent protein kinase A (PKA) were also found. Only one of them (Vedah_comp459933_c0) showed the same behaviour consistently reported in this section (log_2_ RPKM value > 4 at 15 DAI in ‘Picual’ and null in ‘Frantoio’) (Figure 7).

### 3.6. Multi-Task *Verticillium Dahliae* Unigenes Displaying Differential Expression Patterns

**Figure 8:**
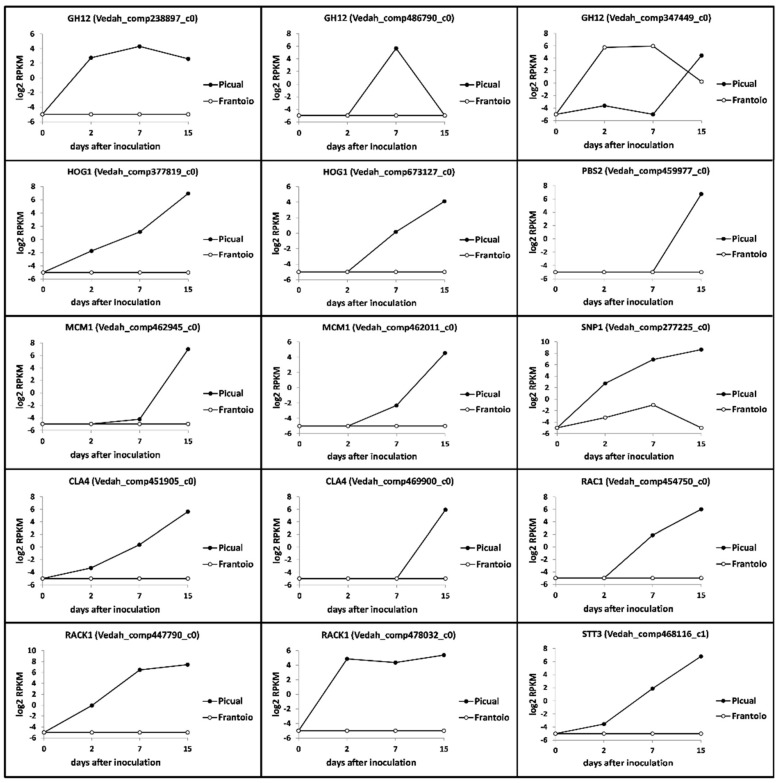
*Verticillium dahliae* unigenes related to phenotypes important during the parasitic phase and differentially expressed in ‘Picual’ and ‘Frantoio’ roots during infection. Samples were analyzed by RNAseq (see the Results section for details). RPKM, Reads Per Kilobase Million.

Several unigenes putatively coding for GH12 (Glycoside hydrolase 12) proteins (virulence factors and pathogen-associated molecular patterns [PAMPs] in oomycetes) were found in the V937-I transcriptome. Three transcripts showed significantly-different expression patterns that are worth mentioning. Two of them did not express at all in ‘Frantoio’ while they did in ‘Picual’, either consistently over time (Vedah_comp238897_c0) or just transiently at 7 DAI (Vedah_comp486790_c0) (Figure 8). Remarkably, one transcript (Vedah_comp347449_c0) behaved distinctly to the other putative GH12, displaying up regulation at 2 and 7 DAI in ‘Frantoio’ while in ‘Picual’ roots was fully repressed until 15 DAI, when a clear induction was observed in contrast to ‘Frantoio’ (Figure 8).

Unigenes coding for another MAPK, high-osmolarity glycerol (HOG1), were also found but only two of them (Vedah_comp377819_c0 and Vedah_comp673127_c0) showed significant expression levels in ‘Picual’, mainly at 15 DAI, and null in ‘Frantoio’ (Figure 8). Interestingly, a transcript (Vedah_comp459977_c0) potentially coding for PBS2 (a MAPK kinase) and that together with HOG1 are involved in a cascade that regulates microsclerotia formation and virulence, was also found to be significantly up regulated in ‘Picual (but not in Frantoio) at 15 DAI (Figure 8). The same behaviour (strong up regulation at late times; 15 DAI) was observed for two unigenes (Vedah_comp442945_c0 and Vedah_comp462011_c0) potentially coding for MCM1, a MADS-box transcription factor performing pleiotropic functions in *V. dahliae* (Figure 8). Several transcripts coding for RAC1 (a small GTPase) and its effector CLA4 have been found in the V937-I transcriptome. Among them, only one unigene for RAC1 (Vedah_comp454750_c0) and two for CLA4 (Vedah_comp451905_c0 and Vedah_comp469900_c0) showed rather similar up regulation patterns from 7 DAI, and only in ‘Picual’ roots (Figure 8). Several unigenes coding for RACK1 (receptor for activated C-kinase) were found from which two of them showed log_2_ RPKM values > 4, one with a consistent pattern along time (Vedah_comp478032_c0) and the other from 7 DAI (Vedah_comp447790_c0), while in ‘Frantoio’ expression was null (Figure 8). Among the transcripts potentially coding for CP1, a member of the SnodProt1 (cerato-platanin protein, CPP) phytotoxin family, some showed log_2_ RPKM values > 4 but with erratic expression patterns over time in ‘Picual’ (no expression in ‘Frantoio; data not shown). However, one of them (Vedah_comp277225_c0) showed a clear up-regulation trend over time, reaching log_2_ RPKM values > 8 at 15 DAI in ‘Picual’ in contrast to the nearly negligible expression observed in ‘Frantoio’ roots (Figure 8). Finally, one transcript (Vedah_comp468116_c0) coding for STT3 (catalytic subunit of the multi-subunit oligosaccharyl transferase, OST) is worth mentioning because, in agreement with the consistent expression pattern summarized here for many unigenes of the V937-I transcriptome, a clear up regulation over time only in ‘Picual’ roots was observed, particularly at late times (15 DAI) (Figure 8).

## 4. Discussion

Results showed that the amount of *V. dahliae* transcripts present in root tissues positively correlated with the VWO susceptibility level of the cultivar. Differences found were outstanding, ‘Frantoio’ roots harboring much lower amounts of V937-I biomass over time compared to that observed in ‘Picual’. Moreover, considering the ratio of ‘Picual’/’Frantoio’ percentage of reads a steady and significant increase of this ratio was also observed over time, reaching a maximum at 15 DAI. At this time-point *V. dahliae* is known to be well established in olive root tissues under artificial inoculation conditions [41]. These results agree with Quantitative Real-Time PCR (Q-RT-PCR) data from previous studies in which the amount of *V. dahliae* DNA in roots was always lower in VWO-tolerant cultivars [41,42,43]. Our results further support this correlation since *V. dahliae* transcriptional activity was significantly higher when the pathogen colonized and infected ‘Picual’ roots. This finding may be relevant in order to explain the differential susceptibility to *V. dahliae* reported for these cultivars, and must be linked to the differential whole transcriptome response earlier reported for the host. Therefore, tolerance of ‘Frantoio’ plants is not only a consequence of a complex process in which numerous plant traits are involved [25], but the result of basal and early pathogen-induced transcriptomic responses in the host hindering pathogen’s biomass proliferation and, consequently, limiting its transcriptional mechanism.

Co-transcriptomic studies of plant-pathogen interactions allow to infer global events taking place in both partners of the interaction. However, these studies are difficult because of the complexity for accurate discrimination between transcripts from the host and the pathogen. Therefore, these approaches are not frequent and have mostly focused on individual genes or gene co-expression networks e.g., [44,45,46,47]. Previously, we conducted a study in which olive (cv. Picual) and *V. dahliae* co-transcriptomes were generated [24]. Later, comparative transcriptomics focused on the differential host responses taking place in olive roots upon *V. dahliae* D pathotype infection, and their correlation with the VWO susceptibility level of olive cultivars was carried out [25]. One of the main results of this later study was that a much higher number of host unigenes were upregulated in ‘Picual’ roots during the early moments of the interaction (up to 7 DAI). In the present study, the number of *V. dahliae* induced unigenes was also demonstrated to be overwhelmingly higher (6 fold) in the susceptible variety. Therefore, both the pathogen (this study) and the host [25] displayed a much higher transcriptional activity (overall up regulation) during a compatible interaction. Data obtained are a reflection of the actual situation taking place in the olive roots: *V. dahliae* proliferating at a much higher rate in ‘Picual’ than in ‘Frantoio’, thereby producing more transcripts and consequently facilitating the detection of low expressed genes in the former. However, the availability of detecting a fair number of unigene transcripts in both cultivars allowed us to perform most specific enriched GO terms analyses, making it possible to observe clear differences between cultivars (Figure 2, Figure 3, Figure 4 and Figure 5 and Figures S1–S3).

According to the expression pattern of *V. dahliae* transcripts observed during the time-course infection process, seven different clusters of expressed unigenes during the root infection were found. Some of the most relevant findings are now discussed. In Group 1 (i.e., expression during the whole sampling period), differences were not only quantitative (the number of *V. dahliae* transcripts whose expression was detected in ‘Picual’ was 11-fold higher compared to ‘Frantoio’) but also qualitative. Remarkably, the pathogen was mainly enriched in transcripts related to cellulose and xylan catabolic processes when colonizing the roots of the tolerant cultivar, and this pattern was consistent over time. In contrast, these most specific enriched GO terms were overexpressed in ‘Picual’ roots only at 15 DAI (group 6; Figure 4). Previously, we identified an olive unigene potentially coding for a Dirigent-like protein involved in lignification that showed a significantly higher basal expression in ‘Frantoio’ than in ‘Picual [25], suggesting that ‘Frantoio’ roots can be more lignified as otherwise reported for the VWO-tolerant cv. Sayali [17]. The lignocellulosic biomass is mainly composed of lignin, cellulose and xylan [48]. Early and consistent induction over time of these *V. dahliae* genes in the interaction with ‘Frantoio’ (but not in ‘Picual’) can be explained by the need to dismantle the higher content of these polymers present in the roots of the tolerant cultivar. The production and involvement of cellulases in pathogenicity and virulence has been demonstrated for different phytopathogenic fungi, including *V. dahliae* e.g., [49,50,51,52]. Gui and co-workers [53] recently reported two GH12 proteins in *V. dahliae* Vd991, VdEG1 and VdEG3, with both cellulase activity required for virulence and elicitor activity that induces host-dependent immunity during *Nicotiana benthamiana* infection. They demonstrated that both proteins acted as PAMPs triggering cell death and PAMP-triggered immunity (PTI), although this function was independent of their enzymatic activity. Ten unigenes coding for GH12 proteins were found in the V-937I transcriptome. Only three of them, potentially coding for xyloglucan-specific endo-β-1,4-glucanase A, displayed significantly-different expression patterns in ‘Picual’ and ‘Frantoio’, although none corresponded to *VdEG1* and *VdEG3*. In fact, *VdEG1* matched to transcript Vedah_comp428223_c0 that only displayed significantly different up regulation at 15 DAI in ‘Picual’ compared to the null expression in ‘Frantoio’ (data not shown). Whether any of these *GH12* genes may play a similar role in the V-937I-olive interaction to that reported by Gui and co-workers [53] is not known. Moreover, the unique discrepant expression pattern (transcript Vedah_comp347449_c0; Figure 8) compared to the other GH12, and to the overall induction observed for pathogenicity- and virulence-related genes in ‘Picual’ in contrast to the null expression in ‘Frantoio’, deserves to be further studied. Another relevant observation when examining group 6 (as well as groups 5 and 7) was the absence of pathogen’s transcripts in ‘Frantoio’ roots. In addition to the above-mentioned late (15 DAI) induction in ‘Picual’ roots of *V. dahliae* genes involved in cellulose and xylan catabolic processes, what may be explained by the pathogen colonizing more lignified regions of the ‘Picual’ roots while progressing upwards during the host colonization process, a number of catabolic processes were also induced at this time-point. Moreover, the conidia formation process was actively up regulated in ‘Picual’ in contrast with ‘Frantoio’, which also correlate with their differential VWO susceptibility level. While penetration and internal root colonization in ‘Frantoio’ is severely hampered, massive entrance of V937-I biomass into ‘Picual’ roots seemed to lead to active conidiation thereby favouring pathogen dissemination through the vascular vessels. The rapid upward spread of *V. dahliae* in the vascular tissue is primarily attributed to conidia translocated by the transpiration stream, a scenario known to take place in trees since long time ago [54]. In fact, D pathotype biomass detection by both microscopy [10] and molecular [36] approaches in aerial organs of ‘Picual’ at early or middle-term times after root inoculation is plausibly explained by conidia production, rapid upwards translocation, and subsequent germination rather than by hyphal proliferation through the vascular system. This scenario is seldom (or never) observed in tolerant cultivars [41,42,43]. To further support the induction of the conidiation process in ‘Picual’, it is worth mentioning the up regulation over time, reaching a log_2_ RPKM value > 6 at 15 DAI, of one transcript potentially coding for STT3 (catalytic subunit of the multi-subunit OST that plays a key role in glycoprotein modification) in ‘Picual’ but not in ‘Frantoio’. Su and co-workers [55] have recently demonstrated that the expression of the *STT3* gene of *V. dahliae* was increased at the stage of conidia germination, and that mycelial growth, sporulation ability and glycoprotein secretion were impaired, as well as germination ratio and virulence decreased in knockout mutants of this gene. Finally, the abundance of most specific enriched GO terms in groups 5, 6 and 7 related to energy generation, cellular response to nutrients and catabolic processes, among others, indicate that in ‘Picual’ roots the pathogen is actively growing and demanding nutrients, likely more available as a consequence of the active host tissue infection process. This situation does not take place in ‘Frantoio’ roots as demonstrated by the negligible amounts of V-937I biomass and the total absent of transcriptional activity, particularly at 15 DAI.

Effective colonization of a host means that a given pathogen is adapted to an ecological niche that provides specific nutrients, sometimes constituting a growth limiting factor for the invader. Moreover, stress conditions must be confronted by the pathogen within the colonized microhabitat (in this case the xylem vessels during most of *V. dahliae*’s parasitic phase), as well as defence responses deployed by the host [56]. In order to assess whether the transcriptome of the D isolate V937-I showed differential responses related to niche adaptation and stress condition depending on the infected olive cultivar a number of unigenes were targeted. Overall, the time-course expression patterns of *V. dahliae* DEGs related to these traits did not show noticeable differences regardless the infected olive cultivar and the ad hoc threshold log_2_ RPKM basal level value set to be considered as significantly relevant. Some of the unigenes here evaluated could also be assigned to other groups, stressing the fact that many of them can be involved in different processes of the parasitic phase of *V. dahliae*. For instance, one of the few remarkable differences found was an unigene coding for SNF1 (high expression in ‘Picual’ at 15 DAI but null expression in ‘Frantoio’). This gene, coding for a sucrose nonfermenting 1 enzyme, seems to act as part of a nutrient signalling pathway controlling both microsclerotial development and pathogenicity [56,57], and thus could also be allocated in our study to the pathogenicity/virulence genes group. *Verticillium dahliae* tomato race 1 mutants in which *SNF1* was disrupted showed no expression of cell wall-degrading enzymes in simulated xylem medium, much lower growth when cultivated in medium amended with pectin and galactose, severe reduction in virulence on tomato and eggplant, and defective early colonization of roots [57]. High induction of this gene in V-937I at late times of the root infection in the susceptible olive cultivar can correspond to nutrient response processes exacerbated in ‘Picual’ as consequence of the massive colonization and degradation of root host tissues. Related to this, the up regulation of two (out of 5) unigenes potentially coding for chorismate synthase (key enzyme in the first branch point intermediate of aromatic amino acid biosynthesis) at 15 DAI in ‘Picual but not in Frantoio’ may also relate to the profuse colonization of the xylem in the susceptible cultivar at this time point, a microhabitat poor in nutrients including low concentrations of amino acids [58]. During the infection process, *V. dahliae* must also confront ROS protective response by the host. Earlier results have revealed that this response takes place in the pathogen (firstly) and the host (at later times) [24]. Moreover, the overall response to ROS stress was clearly stronger in the susceptible cultivar compared to that observed in ‘Frantoio’ roots [25]. In this present study, no remarkable differences were found but the steady up regulation over time (maximum expression reached at 15 DAI) observed for three transcripts potentially coding for PRX1 (a thiol-specific peroxidase) only in ‘Picual’ roots. A PRX1 protein was reported to be abundantly secreted in the xylem sap of hop plants upon infection of *V. nonalfalfae*. Moreover, in planta expression of the coding gene increased with the pathogen’s colonization process, suggesting a role in virulence [59].

None of the examined transcripts potentially coding for known *V. dahliae* effectors and detected in the V-937I transcriptome showed noticeable differences in their expression patterns in the two olive cultivars. An effector role in infecting hosts has been recently suggested for a conserved secretory protein VdCP1 that also showed chitin-binding properties, identified as a member of the SnodProt1 phytotoxin family [60]. These authors reported that *vdcp1* gene displayed high expression along the *V. dahliae* infection process, and also suggested that VdCP1 could protect *V. dahliae* cell wall from enzymatic degradation. Eight unigenes coding for these CPP were found in the V-937I trasncriptome, but only four showed log_2_ RPKM values > 4. Interestingly, one of them displayed an increasing and steady up regulation overtime in ‘Picual’ in contrast to ‘Frantoio’, suggesting a similar role during the infection process of a susceptible olive cultivar to that reported by Zhang and co-workers [60].

Finally, transcripts potentially coding from pathogenicity and virulence showed the same overall differential expression patterns consistently observed between olive cultivars. For instance, the induction at 15 DAI of a transcript coding for a MSB2 protein in ‘Picual’ but not in ‘Frantoio’ is worth mentioning, since a *VdMsb* gene has been implicated in the colonization and penetration processes of cotton and *Arabidopsis thaliana* by *V. dahliae* strain Vd8 [61]. Similarly, transcripts coding for HMG-CoA synthase, CYC8, MAPK1, PKA1, HOG1 and PBS2 were also found to be induced in ‘Picual’ but not in ‘Frantoio’, and always reaching high log_2_ RPKM values at 15 DAI. The link between the expression of genes coding for these proteins and processes such as conidiation, pathogenesis, virulence, microsclerotia formation, etc., has been highlighted in a number of studies, e.g., [62,63,64,65]. Altogether, the high induction of genes involved in these processes when infecting ‘Picual’ roots denotes that colonization and proliferation of the pathogen’s biomass in root tissues of the susceptible cultivar is progressing effectively, and that the host defence responses have been overcome. These events were not observed in ‘Frantoio’ roots. Massive proliferation of the D pathotype in ‘Picual’ roots, including production of microsclerotia on the root surface, has been previously demonstrated by microscopy evidence [10].

## 5. Conclusions

The *V. dahliae* D pathotype transcriptome responds differently depending on the VWO susceptibility level of the cultivar. Differences are not only qualitative along the time-course of the early root colonization process, but mostly quantitative. While outstanding differences were found regarding the biological processes induced in the pathogen, the much larger *V. dahliae* biomass found in roots of ‘Picual’ plants, consequently leading to a much higher transcriptional activity, seems to explain to a large extent the higher aggressiveness exerted over this cultivar and why a susceptible cultivar succumb much faster to the disease. The comparative co-transcriptomic studies performed enabled to holistically disentangle both pathogen and host responses in the olive–*V. dahliae* interaction. VWO susceptibility can thus be explained by the absence of basal and pathogen-induced transcriptomic responses in susceptible varieties [25], which favors the pathogen’s transcriptional mechanism and its massive proliferation, colonization and further dissemination in the host roots.

## Supplementary Materials

The following are available online at https://www.mdpi.com/2073-4425/10/4/251/s1, Figure S1: Group 3 unigenes that present a high mRNA level at 2 days and low at 7- and 15-days post-inoculation. Unigenes were selected using an eightfold change and a 95% of False Discovery Rate; mRNA level in ‘Picual’ (a) and ‘Frantoio’ roots (b); Most specific enriched GO terms of over-expressed genes in *Verticillium dahliae* infecting ‘Picual’ or ‘Frantoio’ roots (c)., Figure S2: Group 4; unigenes that present a high mRNA level at 2- and 15-days but low at 7-days post-inoculation. Unigenes were selected using an eightfold change and a 95% of False Discovery Rate; mRNA level in ‘Picual’ (a) and ‘Frantoio’ roots (b); Most specific enriched GO terms of over-expressed genes in *Verticillium dahliae* infecting Picual or Frantoio roots (c)., Figure S3: Group 5; unigenes that present a high mRNA level at 7 days but low at 2- and 15-days post-inoculation. Unigenes were selected using an eightfold change and a 95% of False Discovery Rate; mRNA level in ‘Picual’ roots (a) and most specific enriched GO terms of over-expressed genes in *Verticillium dahliae* infecting ‘Picual’ roots (b).
