# Supplementary figures and images for: The Transcriptome of Verticillium dahliae Responds Differentially Depending on the Disease Susceptibility Level of the Olive (Olea europaea L.) Cultivar
Source: Genes (Basel). 2019 Mar 27;10(4):251. doi: 10.3390/genes10040251 (PMC6523120; doi:10.3390/genes10040251)

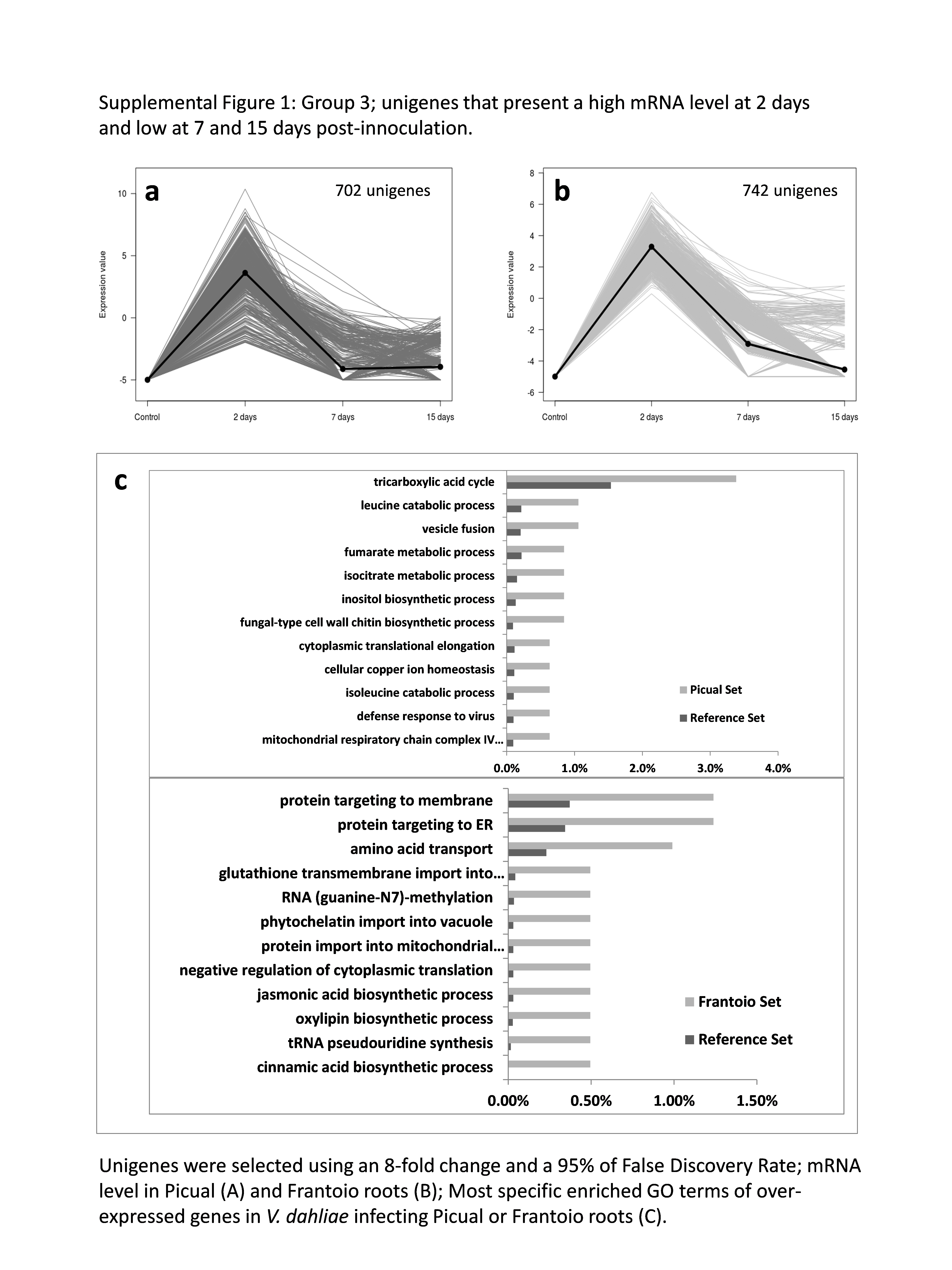

Supplement: Supplementary file 1 [file genes-10-00251-s001.zip › Sup Figure 1 revised.tif]

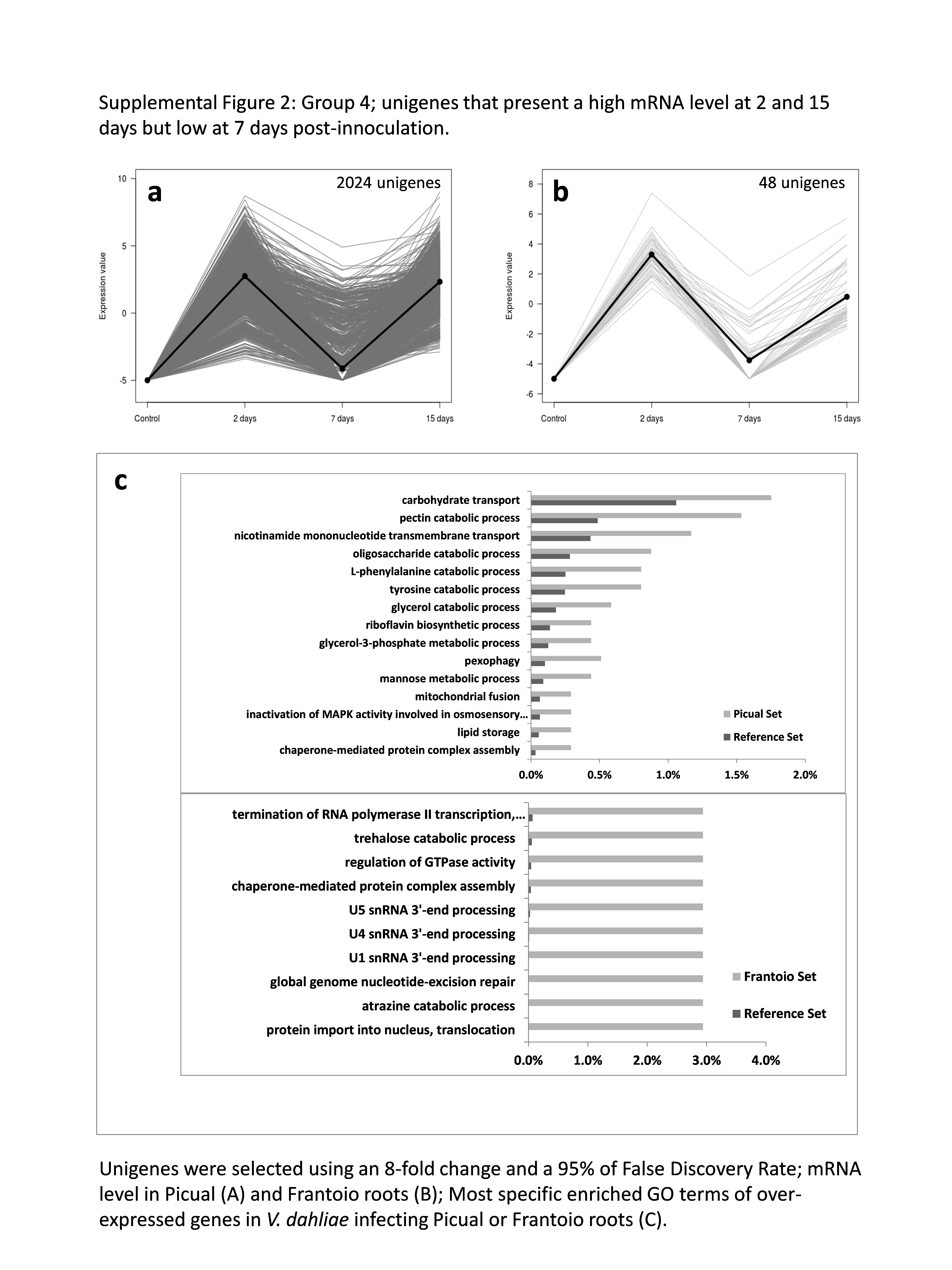

Supplement: Supplementary file 1 [file genes-10-00251-s001.zip › Sup Figure 2 revised.tif]

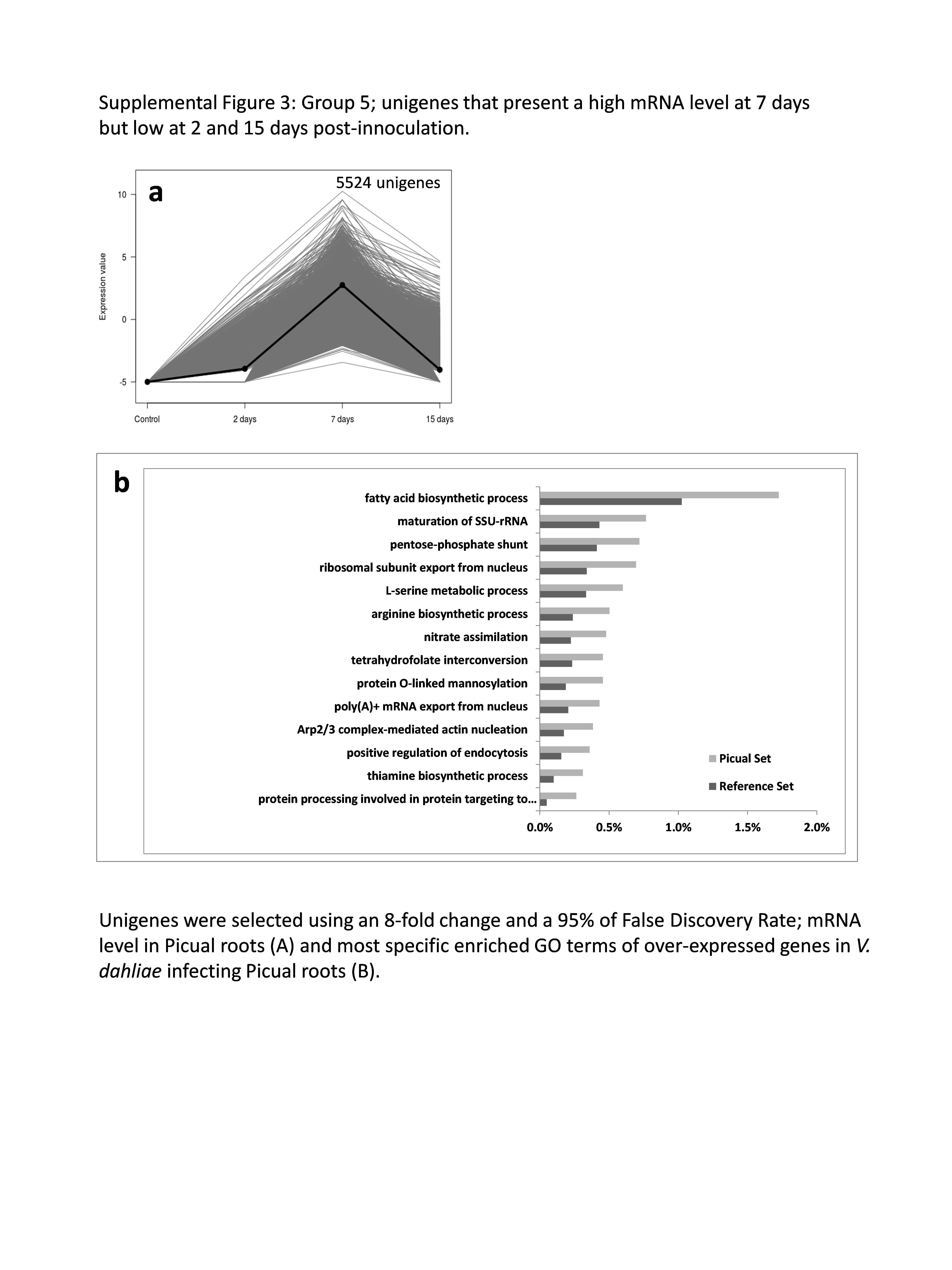

Supplement: Supplementary file 1 [file genes-10-00251-s001.zip › Sup Figure 3 revised.tif]
